# Comparison of Individual Radiosensitivity to γ-Rays and Carbon Ions

**DOI:** 10.3389/fonc.2016.00137

**Published:** 2016-06-13

**Authors:** Grace Shim, Marie Delna Normil, Isabelle Testard, William M. Hempel, Michelle Ricoul, Laure Sabatier

**Affiliations:** ^1^Commissariat à l’Energie Atomique (CEA), DRF/PROCyTOX, Fontenay-aux-Roses, France; ^2^CEA Grenoble, Laboratoire de Chimie et Biologie des Métaux, BIG, DRF, Grenoble, France

**Keywords:** individual radiosensitivity, carbon ions, radiotherapy, relative biological effect, linear energy transfer, isodose effect

## Abstract

Carbon ions are an up-and-coming ion species, currently being used in charged particle radiotherapy. As it is well established that there are considerable interindividual differences in radiosensitivity in the general population that can significantly influence clinical outcomes of radiotherapy, we evaluate the degree of these differences in the context of carbon ion therapy compared with conventional radiotherapy. In this study, we evaluate individual radiosensitivity following exposure to carbon-13 ions or γ-rays in peripheral blood lymphocytes of healthy individuals based on the frequency of ionizing radiation (IR)-induced DNA double strand breaks (DSBs) that was either misrepaired or left unrepaired to form chromosomal aberrations (CAs) (simply referred to here as DSBs for brevity). Levels of DSBs were estimated from the scoring of CAs visualized with telomere/centromere-fluorescence *in situ* hybridization (TC-FISH). We examine radiosensitivity at the dose of 2 Gy, a routinely administered dose during fractionated radiotherapy, and we determined that a wide range of DSBs were induced by the given dose among healthy individuals, with highly radiosensitive individuals harboring more IR-induced breaks in the genome than radioresistant individuals following exposure to the same dose. Furthermore, we determined the relative effectiveness of carbon irradiation in comparison to γ-irradiation in the induction of DSBs at each studied dose (isodose effect), a quality we term “relative dose effect” (RDE). This ratio is advantageous, as it allows for simple comparison of dose–response curves. At 2 Gy, carbon irradiation was three times more effective in inducing DSBs compared with γ-irradiation (RDE of 3); these results were confirmed using a second cytogenetic technique, multicolor-FISH. We also analyze radiosensitivity at other doses (0.2–15 Gy), to represent hypo- and hyperfractionation doses and determined that RDE is dose dependent: high ratios at low doses, and approaching 1 at high doses. These results could have clinical implications as IR-induced DNA damage and the ensuing CAs and genomic instability can have significant cellular consequences that could potentially have profound implications for long-term human health after IR exposure, such as the emergence of secondary cancers and other pathobiological conditions after radiotherapy.

## Introduction

Current radiotherapy regimens use photons or protons for the treatment of a plethora of malignancies. However, as ionizing radiation (IR) of high linear energy transfer (LET) may potentially offer radiobiological advantages over low LET IR due to their inherent physical dose distribution characteristics, cancer radiotherapy is now shifting to the use of high-LET heavier ion species (1). Low LET IR (e.g., X- and γ-rays) deposits exponentially decreasing amounts of energy, as a function of penetration depth in the target material, in a uniform pattern of distribution. High LET IR, such as heavy ions, on the other hand, are characterized by a relatively low entrance dose in the target material, followed by a pronounced sharp maximum dose near the end of their range called the Bragg peak, and energy close to 0 beyond the Bragg peak. This characteristic of high LET IR is useful especially for the treatment of deep-seated tumors in the human body, as it allows a great amount of energy to be precisely localized at the tumor site when it is placed at the Bragg peak, while minimally exposing the surrounding normal tissues (2).

Among various types of heavy ion species considered for radiotherapy, carbon ions are considered to have the most balanced and optimal properties in terms of physical dose distribution and relative biological effectiveness (RBE) along its Bragg peak curve (3). However, carbon ion radiotherapy is not yet widely used, with only a few centers worldwide (six in Asia and two in Europe) that have treated ~13,000 patients (as of December 2013), compared with ~50 active proton therapy centers worldwide that have treated over 105,000 patients (4). Though preliminary clinical data from the existing carbon ion therapy centers suggest favorable results for many of the malignancies that do poorly with conventional radiotherapy (3), further clinical research and development of more carbon ion (and other charged particles heavier than protons) therapy centers in the US and worldwide are hindered by the lack of sufficient clinical evidence of the benefit of carbon ion therapy over conventional radiotherapy that would cost-effectively justify the establishment of such expensive facilities (1). Further investigation is necessary to characterize and understand how carbon ion therapy works in comparison to conventional radiotherapy.

Clinical outcomes of radiotherapy can be significantly influenced by interindividual variations in sensitivity to IR, which is well established to exist in the general population. Highly radiosensitive patients, for instance, may develop early and/or late side effects due to radiation toxicity, while radioresistant patients may receive an insufficient dose of radiation due to dose limitations in current general radiotherapy protocols. However, current radiotherapy and radiation protection protocols do not take into account the individual variations in radiosensitivity, but rather rely on population averages of radiation responses. Refining these protocols to consider individual radiosensitivity, especially the more radiosensitive and cancer-prone, may help to alleviate the detrimental delayed effects of IR (5–7).

In this study, we evaluate individual radiosensitivity following exposure to carbon-13 ions or γ-rays in peripheral blood lymphocytes (PBL) of healthy blood donors using the telomere/centromere-fluorescence *in situ* hybridization (TC-FISH) technique. TC-FISH, which simultaneously stains telomeres and centromeres using peptide nucleic acid (PNA) probes (8), was shown in a recent study in our laboratory (9) to be a cost-effective method that significantly simplifies and improves the “gold standard” dicentric chromosome (DC) assay, which relies on the manual scoring of DCs following Giemsa staining by trained specialists. The radiosensitivity of each analyzed individual in this analysis was ranked based on the estimation of the frequency of IR-induced DNA double strand breaks (DSBs) that either was misrepaired or left unrepaired to form chromosomal aberrations (CAs). For brevity, we refer to these misrepaired or unrepaired DSBs that generate CAs simply as “DSBs” henceforth. Levels of DSBs were estimated from the scoring of CAs visualized with TC-FISH, including dicentrics, centric and acentric rings, and acentric fragments (with 0, 2, or 4 telomeres). We demonstrated in our previous article (9) that this modified scoring technique provides improved sensitivity compared with the classical DC analyses. Additionally, as presented in this same paper, we developed a novel automated system (TCScore) that can perform these TC-FISH analyses with the same efficacy as manual scoring, but in a fraction of time; this improved, automated approach will open up new horizons for the assessment of genotoxic risk and for biological dosimetry, particularly for low doses.

We examine radiosensitivity at the dose of 2 Gy, a routinely administered dose during fractionated radiotherapy (10, 11), and at other doses (0.2–15 Gy), to represent hypo- and hyperfractionation doses. As we are particularly interested in comparing the levels of biological effect (misrepaired or unrepaired DNA DSBs generating CAs in this case) at a particular dose of carbon irradiation compared with the same dose of γ-irradiation (isodose effect), we also define a quality we term “relative dose effect” (RDE). This ratio is advantageous as it allows for simple comparison of dose–response curves.

## Results

### Individual Radiosensitivity Following Exposure to 2 Gy of γ-Rays

**Figure 1:**
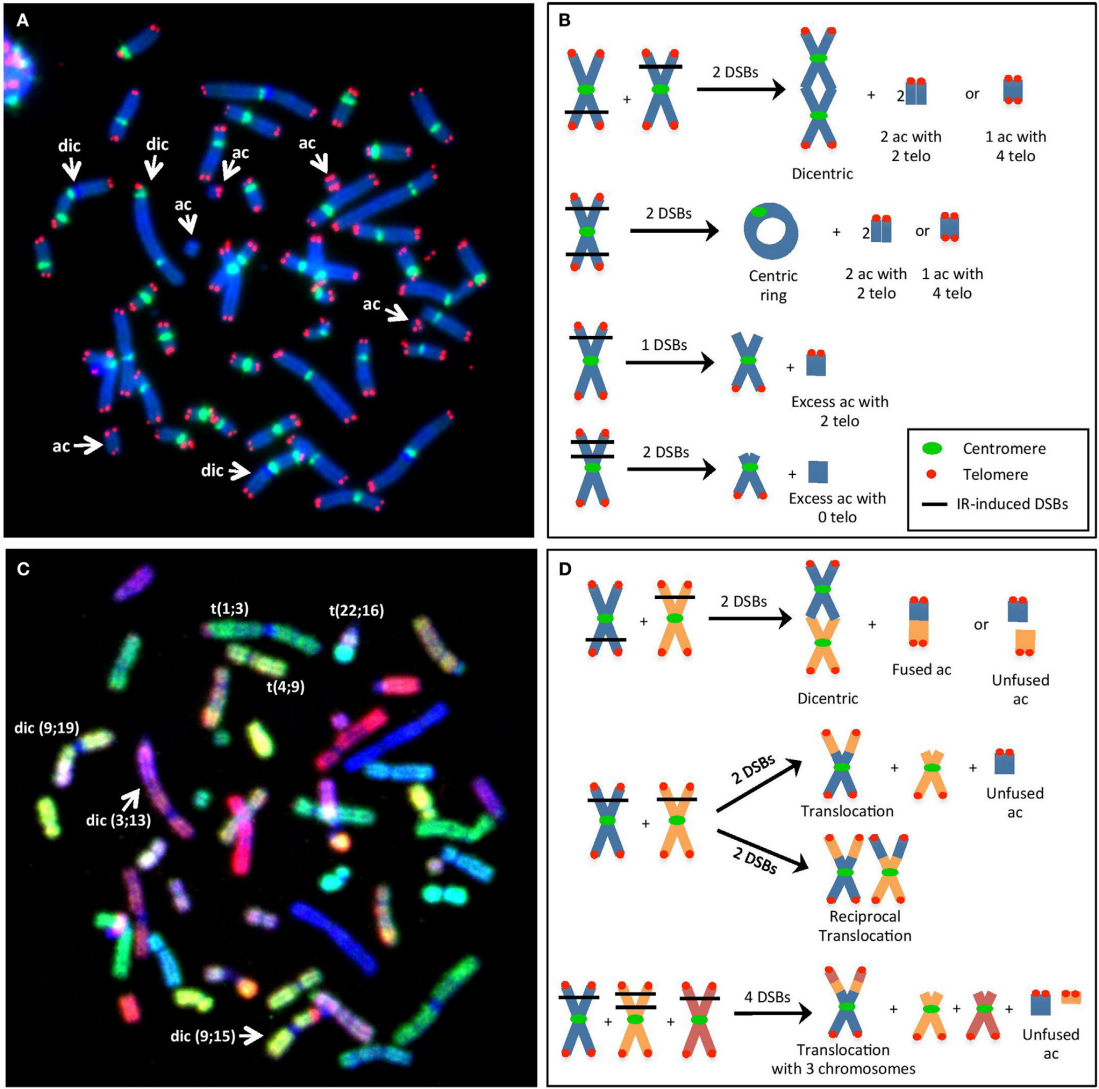
**(A)** Visualization of IR-induced dicentric chromosomes (dic) and other chromosomal aberrations (CAs), such as acentric fragments (ac), using telomere/centromere-fluorescence *in situ* hybridization (TC-FISH). This image shows 3 dic and 5 ac [3 with 4 telomeres (telo), 1 with 2 telo, 1 with 0 telo]. **(B)** Examples of the method used to estimate the number of IR-induced DSBs per cell (i.e., misrepaired or unrepaired DSBs that generated CAs) using TC-FISH. A dic or a centric ring with an ac containing four telo was considered as two DSBs. Excess ac with two telomeres was considered as resulting from one DSB that failed to rejoin (terminal deletion). Excess ac with 0 telomeres were considered as resulting from 2 DSBs (interstitial deletion). Note that these sample images of chromosomes are not an analysis of **(A)**, and lines denoting DSBs from IR interactions are not necessary from traversal with the same IR track. **(C)** Visualization of IR-induced translocations using M-FISH. This image shows the same metaphase as in **(A)**. Each chromosome involved in the dic and ac can be identified. Furthermore, three additional translocations can be observed that was not able to be visualized using the TC-FISH technique. **(D)** Examples of the method used to estimate the number of IR-induced DSBs (i.e., misrepaired or unrepaired DSBs that generated CAs) using M-FISH. Dic or translocations involving two chromosomes often involve two DSBs, whereas more complex rearrangements with three chromosomes may involve four DSBs. Note that these sample images of chromosomes are not an analysis of **(C)**, and lines denoting DSBs from IR interactions are not necessary from traversal with the same IR track.

**Figure 2:**
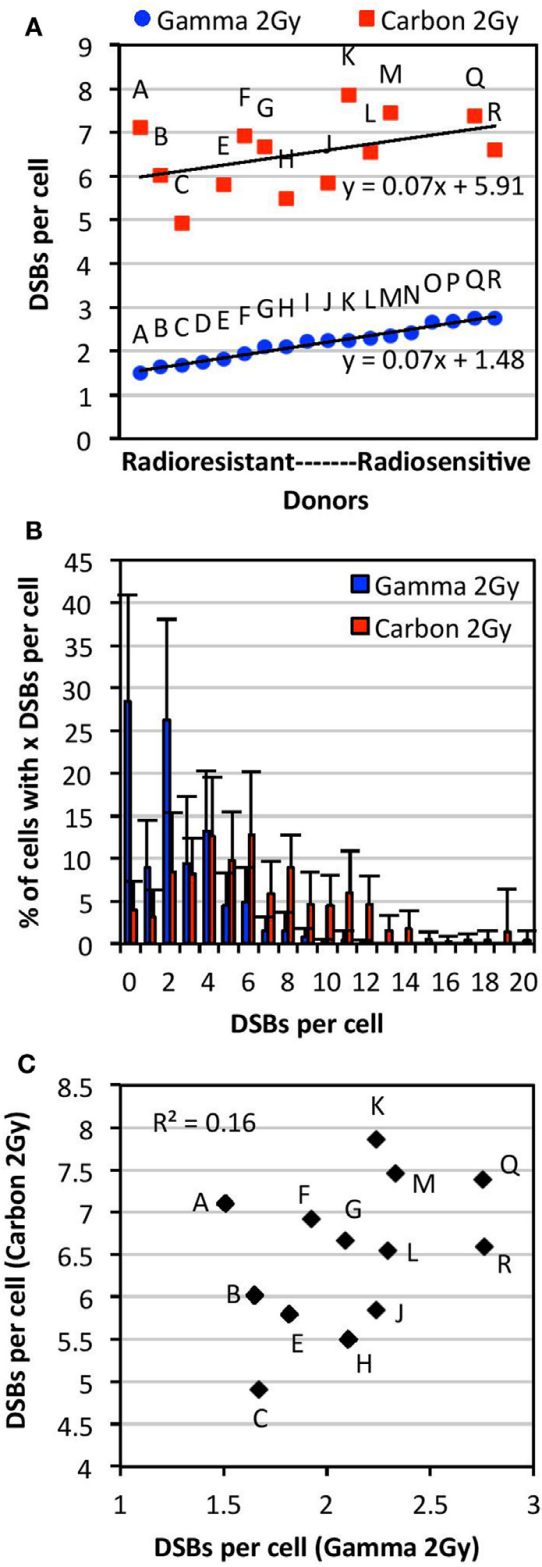
**Comparison of individual radiosensitivity following exposure to 2 Gy of either carbon-13 ions (75 MeV/u; LET ~36.5 keV/μm at the plateau region of the Bragg peak curve) or γ-rays**. Individual radiosensitivity was evaluated in peripheral blood lymphocytes (PBL) of healthy blood donors in cells undergoing first mitosis at 60 h postirradiation; radiosensitivity of each individual was ranked using the TC-FISH technique based on the estimation of the frequency of IR-induced DNA DSBs (i.e., misrepaired or unrepaired DSBs that generated CAs), estimated as shown in Figure 1B. **(A)** Ranking of individual radiosensitivity to 2 Gy of carbon ions and γ-rays. Individuals were designated as Donors A (“radioresistant”) through R (“radiosensitive”) based on the order of increasing radiosensitivity following γ-irradiation. **(B)** Distribution of the number of DSBs per cell for each type of IR for all donors analyzed. **(C)** No correlations between individual radiosensitivity following *in vitro* exposure to 2 Gy of carbon ions and γ-rays (*R*^2^ = 0.16).

Individuals in this cohort of 18 healthy blood donors were first ranked in the order of increasing radiosensitivity based on the mean number of IR-induced DSBs (i.e., misrepaired or unrepaired DSBs that generated CAs) per cell following *in vitro* exposure of isolated PBL to 2 Gy of low LET γ-rays. The mean number of DSBs per cell was calculated based on the scoring of CAs following TC-FISH staining, as described in Figures 1A,B, in cells undergoing first mitosis at 60 h postirradiation. As shown in Figure 2A, individuals were designated as Donors A through R in this order of “radioresistant” to “radiosensitive” donors. We use this ranking throughout the study as the definition of each of these donors’ radiosensitivity.

Following exposure to a dose of 2 Gy of γ-irradiation, there was a range of ~1.5–2.8 DSBs per cell (1.8-fold difference), and a mean of 2.17 DSBs per cell in the PBL samples. Comparison of data obtained from samples irradiated on different dates and analyzed by different individuals showed no significant differences in the measurement of the mean number of DSBs per donor (*p* > 0.05). Donors classified as more radiosensitive harbored more DSBs per cell, with a wider range of distribution of DSBs per cell, compared with the more radioresistant donors (Figure S1 in Supplementary Material). For example, the mean of the range of DSBs per cell in radioresistant donors (Donors A through F) was found to be 9.0 compared with 12.5 in radiosensitive donors (Donors M through R). This indicates the presence of more IR-induced damage in radiosensitive donors compared with radioresistant donors following exposure to a dose of 2 Gy of γ-rays.

No correlations were observed between this radiosensitivity and levels of spontaneous or IR-induced apoptosis (0–6 Gy; data not shown). Furthermore, no correlations were found (*R*^2^ = 0.045; data not shown) between radiosensitivity to 2 Gy of γ-irradiation and the susceptibility to IR-induced apoptosis in the T4-EM subpopulation (measured as the slope of IR-induced apoptosis in T4-EM lymphocytes between the doses of 0 and 6 Gy of γ-irradiation), as previously described (12). Radiosensitivity may be moderately correlated with interindividual variability in the induction of global γH2AX fluorescence at 30 min postirradiation (*R*^2^ = 0.595), but not at later time points postirradiation (3–24 h); global γH2AX fluorescence data of this cohort of PBL were previously published (13).

### Individual Radiosensitivity Following Exposure to 2 Gy of Carbon Ions

Individual radiosensitivity following *in vitro* exposure to 2 Gy of high LET carbon-13 ions (75 MeV/u; LET ~36.5 keV/μm at the plateau region of the Bragg peak curve) was measured in PBL of 13 of the healthy blood donors analyzed for γ-irradiation above in cells undergoing first mitosis at 60 h postirradiation.

As shown in Figure 2A, interindividual differences in radiosensitivity was also observed following carbon irradiation, a range of ~5–8 DSB per cell was measured (1.6-fold difference), and a mean of 6.45 DSBs per cell in the PBL samples. As with γ-irradiation, radiosensitivity was not correlated with apoptosis and global γH2AX fluorescence (data not shown). Based on the ranking of increasing radiosensitivity following carbon irradiation, we find that the more radiosensitive donors to carbon irradiation harbored more DSBs per cell compared with the more radioresistant donors (Figure S2 in Supplementary Material); for example, the mean of the range of DSBs per cell in radioresistant donors (Donors C, H, E, and J) was found to be 14.8 compared with 19.8 in radiosensitive donors (Donors F, A, Q, M, and K).

### No Correlations between Radiosensitivity to 2 Gy of γ-Rays and Carbon Ions

Comparison of radiosensitivity to carbon irradiation and γ-irradiation at the dose of 2 Gy showed a different order of increasing radiosensitivity within this cohort, as illustrated in Figure 2A. Indeed, the order of low to high radiosensitivity as classified according to 2 Gy of γ-irradiation did not hold for carbon irradiation following exposure to the same dose (Figure 2A). This indicates that donors are not equally sensitive to different types of IR. Interestingly, though the ranking of radiosensitivity to carbon ions and γ-rays was different within this cohort, the trend lines for radiosensitivity to each type of IR (plotted in the order of increasing radiosensitivity to γ-rays) were parallel, both with a slope of 0.07. Notably, a high intracellular variability of IR-induced DSB among cells of the same donor was observed (data not shown). Intracellular variations following carbon irradiation were generally found to be larger than those following γ-irradiation. This may be expected due to the non-uniform spatial distribution of IR-induced DNA damage following heavy ion irradiation. A modest correlation was found between the dispersion of DSBs per donor (95% confidence interval) following γ- and carbon irradiation (*R*^2^ = 0.51). As expected, carbon irradiation causes more dispersion in the number of DSBs induced per cell compared with γ-irradiation, with carbon ranging to up to 20 DSBs per cell and γ-rays ranging up to 12 DSBs (Figure 2B). This indicates that carbon irradiation causes a larger range of DSBs per cell and more IR damage that is less repaired compared with γ-rays. As shown in Figure 2C, we find that there are no correlations between radiosensitivity to carbon ions and γ-rays at the dose of 2 Gy (*R*^2^ = 0.16).

### RDE Factor of 3 after 2 Gy Irradiation Using Both TC-FISH and M-FISH Techniques

In this study, as we are particularly interested in the differences in the effectiveness of induction of DSBs (i.e., misrepaired or unrepaired DSBs that generated CAs) by carbon irradiation compared with γ-irradiation at a given dose (isodose effect), we define a new ratio, termed RDE, calculated simply by dividing the mean DSBs per cell determined using TC-FISH following exposure to carbon ions by that following exposure to the same dose of γ-rays. This ratio differs from the usual metric RBE (defined as the ratio of doses that produce an iso-effect) and is advantageous, as it allows for simple comparison of dose–response curves.

At the dose of 2 Gy, the mean number of DSBs per cell was found to be 2.17 DSB per cell after γ-irradiation (18 donors, as described in Section “Individual Radiosensitivity Following Exposure to 2 Gy of γ-Rays”) and 6.45 DSB after carbon irradiation (13 donors, as described in Section “Individual Radiosensitivity Following Exposure to 2 Gy of Carbon Ions”). Therefore, the RDE of carbon ions was determined to be ~3 times that of γ-rays at the dose of 2 Gy using TC-FISH. The RBE at 2 Gy was found to be 2.6.

Relative dose effect results were confirmed using M-FISH analysis of chromosomal rearrangements, visualized as illustrated in Figure 1C. The number of DSBs per cell using M-FISH analysis was calculated, as illustrated in Figure 1D. At the dose of 2 Gy, M-FISH analyses in four donors (Donors A, C, L, and R) indicated 3.26 DSBs per cell after γ-irradiation and 9.81 DSBs per cell following carbon irradiation. As M-FISH is a more detailed analysis of chromosomal damage compared with TC-FISH (since M-FISH allows analysis of translocations, which are not visible with TC-FISH), it is expected that more DSBs per cell be calculated using M-FISH than using TC-FISH. However, as both techniques give an RDE factor of 3 at the dose of 2 Gy, the determination of RDE factor of carbon compared with γ-rays is independent of the method of scoring chromosomal damage. Thus, TC-FISH and M-FISH can be considered to be two alternative approaches for scoring chromosomal damage.

Based on these results, we propose that the TC-FISH technique is more practical for rapid assessment of genotoxic risk and for radiation dosimetry, as M-FISH is both expensive and time consuming in terms of hybridization technique and analysis compared with TC-FISH.

### RDE at Other Doses: High RDE at Low Doses

To determine RDE at other doses, we compare mean DSBs per cell determined using TC-FISH following exposure to a range of doses (0.2–15 Gy) of carbon ions and γ-rays in a subset of the PBL of the healthy blood donors analyzed above. For γ-irradiation at all doses except for 2 Gy (which is the average of 18 donors; data in Figure 2A), the mean DSBs per cell represent the average of six donors (Donors C, F, H, J, K, and O). For carbon irradiation at all doses except for 2 Gy (which is the average of 13 donors; data in Figure 2A), the mean DSBs per cell represent the average of four donors (Donors G, H, K, and M).

**Figure 3:**
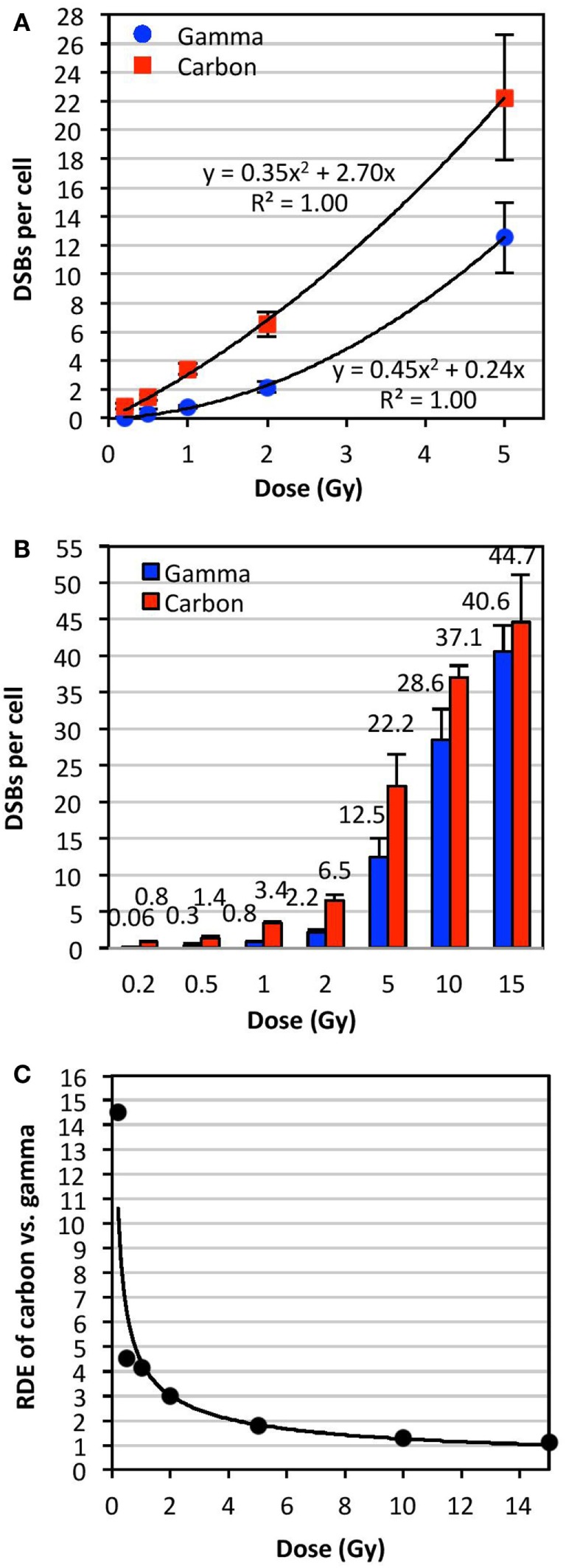
**Relative dose effect (RDE) of carbon-13 ions (75 MeV/u; LET ~36.5 keV/μm at the plateau region of the Bragg peak curve) versus γ-rays at various doses**. We define RDE to be the ratio of biological effect at a given dose (isodose effect). The mean number of DSBs per cell was determined using TC-FISH as illustrated in Figure 1B, and dose–response curves were plotted for doses of up to **(A)** 5 Gy and **(B)** 15 Gy. The mean DSBs per cell for all donors analyzed are indicated above each bar in **(B)**. Error bars represent the SD of the frequencies of DSBs per cell among the donors. **(C)** RDE of carbon ion versus γ-rays as a function of dose.

Figure 3A shows a plot of the dose (0–5 Gy) of γ- or carbon irradiation and the mean number of DSBs per cell averaged for all donors analyzed. This plot indicated second order polynomial trends between the doses of 0 and 5 Gy for both IR types. This plot was expanded to doses of up to 15 Gy in Figure 3B, which shows data for the frequency of DSBs per cell (averaged for all donors analyzed) at each dose with the exact mean indicated above each bar. Error bars in Figures 3A,B represent the SD of the frequencies of DSBs per cell among the averaged donors, illustrating interindividual variations in radiosensitivity at various doses. RDE factors shown in Figure 3C were calculated by dividing the mean DSBs per cell following a dose of carbon irradiation by the mean DSBs per cell following the same dose of γ-irradiation (values shown in Figure 3B). The RDE factor is dose dependent, with high RDE factors at low doses (0.2 and 0.5 Gy), and an RDE factor approaching 1 at high doses (10 and 15 Gy).

## Discussion

In this study, we demonstrate that following *in vitro* irradiation with carbon ions or γ-rays at the dose of 2 Gy, a routinely administered dose during fractionated radiotherapy (10, 11), interindividual differences in radiosensitivity (measured in terms of misrepaired or unrepaired IR-induced DNA DSBs that led to the formation of CAs) exist in healthy individuals. In other words, a given dose of IR can induce a wide range of DNA damage among healthy individuals, with highly radiosensitive individuals harboring more IR-induced damage in the genome than radioresistant individuals following exposure to the same IR dose. These results could have important clinical implications as IR-induced DNA damage and the ensuing CAs and genomic instability can have significant cellular consequences that could potentially have profound implications for long-term human health after IR exposure, such as the emergence of secondary cancers and other pathobiological conditions after radiotherapy (14–16). A fast and reliable clinical method to measure radiosensitivity of cancer patients and/or predict radiotherapy toxicity (especially to identify hyper-radiosensitive individuals) would permit personalized radiotherapy treatment; however, such a method still remains to be established (17–20).

It is well established that radiosensitivity is closely linked to intrinsic, genetically determined differences in cellular responses to IR-induced damage, particularly the repair of DNA DSBs (7). In our previously published paper (13), we have demonstrated, in this same cohort of PBL from healthy individuals, a high level of interindividual variability in the induction and kinetics of γH2AX, an important DNA damage response (DDR) protein that facilitates the efficient repair of DSBs, following γ-irradiation; this variability, measured using global immunofluorescence microscopy and confirmed with flow cytometry, was found to increase with dose and diminish with repair time, in accordance with previously published observations (21–24). This finding supports the notion that these individuals vary in their DDR capacities. However, in this study, we show a moderate correlation between radiosensitivity and global γH2AX fluorescence at 30 min postirradiation (*R*^2^ = 0.595), but no correlations at later time points postirradiation (3–24 h). These moderate to lack of correlations between radiosensitivity and γH2AX levels could be due to the rapid time-dependent changes in γH2AX levels postirradiation. Furthermore, the lack of correlation that we have observed between sensitivity to carbon and γ-irradiation may indicate that individuals may not be equally capable of repairing the different types of DNA damage induced by low LET and high LET IR. Indeed, high LET IR causes more *clustered* DNA DSBs and higher frequencies of complex chromosomal aberrations (CCAs) that may be less likely to be repaired correctly compared with equivalent doses of low LET IR (25–29).

Our results demonstrate that the yield of IR-induced DSBs fits a polynomial curve very close to linearity, in agreement with previous reports of upward curvature, especially following high LET IR (29, 30). We showed that RDE is dose dependent, with high RDE at low doses (0.2 and 0.5 Gy), and approaching 1 at high doses (10 and 15 Gy). This may indicate that the biological effectiveness of carbon at low doses, such as in surrounding tissue of the primary site of irradiation, may be significantly underestimated: IR exposure may be more harmful than expected. On the other hand, at very high doses per fraction, such as in hypofractionated radiotherapy schemes, biological effectiveness may be significantly overestimated. These results may be important to consider for carbon radiotherapy.

In this study, we have found that TC-FISH and M-FISH are two complementary methods for the scoring of DSBs and RDE determination at the dose of 2 Gy, as carbon ions caused three times more DSBs per cell compared with γ-irradiation with both techniques with this dose. We have recently further improved the speed of the TC-FISH technique and analysis in our laboratory with the development of a semi-automated software (TCScore) that is able to detect IR-induced CAs (dicentrics, rings, acentrics with 4, 2, 0 telomeres) with the same efficacy as manual scoring in a fraction of time (9). This software provides automated analysis of three-channel (RGB) images (red, green, and blue channels containing telomere, centromere, and DAPI DNA staining information, respectively) split into their individual channels by any image processing software (e.g., Image J) and generates an intuitive and interactive report of CA classes that can be reviewed and corrected in batches by an investigator. This improved, automated approach will open up new horizons for the assessment of genotoxic risk for clinical uses (e.g., radiosensitivity assessment before radiotherapy) and for biological dosimetry following accidental exposure, particularly for low doses (9, 31). However, in order for these techniques to be used in the clinics for determining intrinsic individual radiosensitivity, analyses of a larger cohort of healthy individuals are needed to well establish the degree of variations within the whole human population.

Meanwhile, the M-FISH technique has been shown to be a powerful tool for detailed analyses of translocations and CCAs in the whole genome at very low to high doses of IR exposure, as it allows all chromosomal homolog pairs to be differentiated (32, 33). It was shown to be sensitive enough to detect translocations and other CAs at doses as low as 0.1 Gy of low LET IR (34). Though the long-term stability of translocations and the usefulness of this technique was recently validated (35), M-FISH analysis is laborious, time consuming (~5 days to obtain results), and expensive; standardization and automation will be key to improving the practical significance of FISH-based translocation assays. Furthermore, the frequencies of translocations at baseline and their persistence postirradiation at various doses, as well as potential interindividual variability in their levels, need to be further characterized, especially in the low dose range (36). Such data would be valuable for studying the long-term health risk of IR exposure and may generally contribute to understanding the link between CAs and human diseases and cancer (37).

In conclusion, it is evident that individual radiosensitivity exists among healthy individuals following irradiation with carbon ions and γ-rays, and individuals may not be equally sensitive to different types of IR. Furthermore, the RDE of carbon compared with γ-rays could be dose dependent, illustrating the complexity of the biological responses to IR. We propose that the calculation of IR-induced DSBs (i.e., misrepaired or unrepaired DSBs that generated CAs) using TC-FISH may be a sensitive and reliable approach to measuring individual radiosensitivity. The ability to rank and predict individual radiosensitivity has a wide range of real-world applications, as it directly impacts the formulation of cancer treatment strategies and the establishment of radiation protection guidelines. Refining radiotherapy and radiation protection protocols to consider individual radiosensitivity, especially the more radiosensitive and cancer-prone, may help to alleviate the detrimental delayed effects of IR.

## Materials and Methods

### Cell Culture

Peripheral blood lymphocytes used in this study were isolated from the whole blood of 18 healthy blood donors (with negative viral status) from the Center of Blood Transfusions using the standard Ficoll isolation technique. Individuals included in this cohort were selected from a larger cohort of 63 individuals along the range of radiosensitivity measured previously based on the induction of IR-induced apoptosis (38); all analyses, however, were performed blindly. After isolation, lymphocytes were frozen in liquid nitrogen (−196°C) until use. Lymphocytes were unfrozen 24 h before irradiation and incubated at 37°C in an atmosphere of 5% CO_2_ in RPMI 1640 medium (Gibco) supplemented with 20% fetal bovine serum (FBS; Eurobio) and antibiotics (penicillin/streptomycin; Gibco).

### Irradiation

Peripheral blood lymphocytes were irradiated at various doses at room temperature (RT) with γ-rays from a Cesium-137 source at the CEA Fontenay-aux-Roses, France (dose-rate of 2 Gy/min).

Carbon-13 (^13^C^6+^) irradiations were performed on the Grand Accélérateur National d’Ions Lours GANIL (Caen, France) D1 high energy line (IRRABAT beam line) with energy of 75 MeV/u; details of dosimetry and other specifications were previously published (39). Lymphocytes were irradiated in small tubes with a glass wall of 2 mm thickness. Samples were irradiated at the plateau region of the Bragg peak curve; the mean LET at the sample was estimated to be ~36.5 keV/μm. The dosimetry was realized with the assistance of CIMAP–CIRIL physicists using a Faraday cup and an X-ray detector (5 μm stainless steel foil and photomultiplier). The photons emitted after traversal of the foil by the accelerated ions were counted, and a correlation at low fluences/doses was established with the real ion tracks measured on CR39 tracks detectors (C_12_H_18_O_7_)_n_. After exposure to the beam, the ion tracks in the CR39 were chemically etched for 8–12 min in 12 N KOH at 80°C. Several microscope fields were photographed using an Olympus Vanox-S, ×100, equipped with a Cohn Pieper FK-7512-Q video camera. The tracks were then counted using a homemade image analysis application from the Aphelion^®^ software. X-ray detector doses were also subsequently correlated with the doses measured with an ionizing chamber (Unidos 23332 or 23344, PTW Freiburg, Germany, depending on the ion atomic number and its track length) for further verification of the dose/fluence ratio. The ionizing chamber was not used as reference dosimeter for the sample irradiations, since it was designed for measuring photon fluxes (utilized in radiotherapy).

### Chromosome Preparation, Staining, and Image Acquisition

Peripheral blood lymphocytes were cultured for 60 h postirradiation, and metaphase preparations were performed using standard procedures (40). Slides with metaphase spreads were stored in −20°C until use, and were unfrozen and left at RT overnight before use.

For TC-FISH analysis, telomeres and centromeres were stained, as previously described (9) using telomere-specific Cyanine3-labeled PNA probes and centromere-specific FITC-labeled PNA probes (both from Panagene, Daejon, South Korea).

For M-FISH analysis, slides were hybridized with a 24XCyte mFISH kit (MetaSystems Altlussheim, Germany) according to the protocol recommended by the manufacturer.

After counterstaining of the DNA with 4′,6-diamidino-2-phenylindole (DAPI), slides were mounted with coverslips with PPD (1 mg/mL *p*-phenylenediamine-90% glycerol-10% PBS) and stored in a dark box at −4°C until automated image acquisition using the MetaSystems AutoCapt software. Images of metaphase cells were acquired with a charge-coupled device camera (Zeiss, Thornwood, NY, USA) coupled with a Zeiss Axioplan microscope. CAs were scored manually using the MetaSystems ISIS software.

### Analysis of Chromosomal Aberrations

In this study, radiosensitivity was measured based on the mean number of IR-induced DSBs per cell (i.e., misrepaired or unrepaired DSBs that generated CAs) following TC-FISH staining (Figure 1A) in cells undergoing first mitosis at 60 h postirradiation. DSBs were calculated based on the manual scoring of CAs, as described in Ref. (9) and in Figure 1B. Based on the frequencies of dicentrics, centric and acentric rings, and acentric fragments (with 0, 2, or 4 telomeres), a precise estimate of the number of IR-induced DSBs that gave rise to the CA can be calculated at the studied doses, as illustrated in Figure 1B. Generally, a dicentric or a centric ring with an acentric fragment containing four telomeres are considered as two DSBs; excess acentric fragments with two telomeres are considered as resulting from one DSB (terminal deletion); and excess acentric fragments with 0 telomeres are considered as resulting from two DSBs (interstitial deletion).

Following M-FISH staining (Figure 1C), the number of DSBs per cell can be calculated based on the visualization of chromosomal rearrangements (that are not visible with TC-FISH). Examples are illustrated in Figure 1D; in general, dicentrics and translocations involving two chromosomes often involve two DSBs, whereas more complex rearrangements with three chromosomes may involve four DSBs.

## Author Contributions

GS analyzed and interpreted the data and drafted the manuscript. MN, IT, and MR worked to design, acquire, and analyze the data. WH aided in the interpretation of the data and edited the manuscript. LS directed the course of this study. All authors provided critiques for the content of the manuscript, approved of the final version of the manuscript, and attest to the accuracy and integrity of this work.

## Conflict of Interest Statement

The authors declare that the research was conducted in the absence of any commercial or financial relationships that could be construed as a potential conflict of interest.
