# Supplementary material for: Comparison of Individual Radiosensitivity to γ-Rays and Carbon Ions
Source: Front Oncol. 2016 Jun 13;6:137. doi: 10.3389/fonc.2016.00137 (PMC4904030; doi:10.3389/fonc.2016.00137)
Supplement: Supplementary file 1 [file Data_Sheet_1.DOCX]

Supplementary Material

Comparison of individual radiosensitivity to gamma-rays and carbon ions

Grace Shim, Marie Delna Normil, Isabelle Testard, William M. Hempel, Michelle Ricoul, Laure Sabatier

*** Correspondence:** Laure Sabatier, laure.sabatier@cea.fr

# Supplementary Figures and Tables

## Supplementary Figures

Supplementary Figure 1. (A) Inter-individual differences in radiosensitivity following *in vitro* exposure to 2 Gy of γ-rays. Radiosensitivity was measured based on the mean number of DSBs (i.e., misrepaired or unrepaired DNA DSBs generating CAs) per cell, calculated based on TC-FISH data as described in Figure 1B, in PBL isolated from whole blood of 18 healthy individuals, in cells undergoing first mitosis at 60 hours post-irradiation. (B) Distribution of the number of DSBs per cell for each donor.

Supplementary Figure 2. (A) Inter-individual differences in radiosensitivity following *in vitro* exposure to 2 Gy of high-LET carbon-13 ions (75 MeV/u; LET ~36.5 keV/µm at the plateau region of the Bragg peak curve). Radiosensitivity was measured based on the mean number of DSBs (i.e., misrepaired or unrepaired DNA DSBs generating CAs) per cell, calculated based on TC-FISH data as described in Figure 1B, in PBL isolated from whole blood of 13 healthy individuals, in cells undergoing first mitosis at 60 hours post-irradiation. (B) Distribution of the number of DSBs per cell for each donor.
